# Appendicitis Secondary to Trauma following a Camel Kick: Case Report and Review of Literature

**DOI:** 10.1155/2021/6667873

**Published:** 2021-01-07

**Authors:** Ali Toffaha, Omer Al-Yahri, Zainab Hijawi, Saif Al-Mudares, Mohannad Al-Tarakji, Fakhar Shahid, Syed Muhammad Ali

**Affiliations:** ^1^Department of General Surgery, Hamad Medical Corporation, Doha, Qatar; ^2^Department of Acute Care Surgery, Hamad Medical Corporation, Doha, Qatar; ^3^Department of Psychiatry, Hamad Medical Corporation, Doha, Qatar

## Abstract

**Introduction:**

Independently, trauma and appendicitis are two of the most common conditions in surgical practice. Rarely, both conditions may coexist, which raises the controversy whether it is merely a coincidence or trauma may lead to acute appendicitis. *Presentation of Case*. We report a case of acute appendicitis after blunt abdominal trauma caused by a camel hoof kick to the abdomen in a young man and discuss the potential underlying pathophysiologic mechanisms with review of the pertinent literature.

**Conclusions:**

Blunt abdominal trauma caused by a camel kick to the abdomen requires a close observation of the patients. A camel kick may increase intra-abdominal pressure and cause internal organ injury including the appendix. Therefore, acute appendicitis should be considered in differential diagnosis in any patient with abdominal pain resembling appendicitis following blunt abdominal trauma.

## 1. Introduction

Appendicitis is one of the most common surgical conditions affecting about 7% of people during their lifetime [1]. The etiology of acute appendicitis is multifactorial, with luminal obstruction being considered the major cause [1]. Blunt abdominal trauma (BAT) has been infrequently reported as a possible cause for acute appendicitis; however, most of the reported cases were in pediatric age group (Table 1). Herein, we report a rare case of acute appendicitis after blunt abdominal trauma caused by a camel hoof kick to the abdomen.

## 2. Case Presentation

A 35-year-old Bangladeshi man presented to the emergency department at Hamad Medical Corporation, Doha, Qatar, with two-day history of progressive right lower abdominal pain, associated with four times vomiting and loss of appetite. He was doing completely well but developed these symptoms few hours after a strong direct camel kick on his right abdomen. He did not have any urological symptoms, nor any comorbidities, and his systemic review was unremarkable.

**Figure 1:**
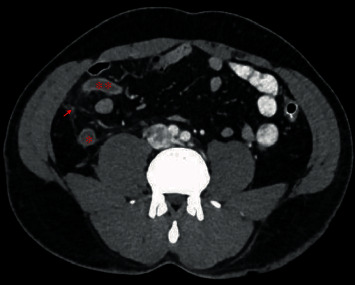
Abdomen CT scan with IV and oral contrast showing dilated appendicular tip (^∗∗^) and base (^∗^), with periappendiceal fat stranding (arrow) and enhancing wall.

**Figure 2:**
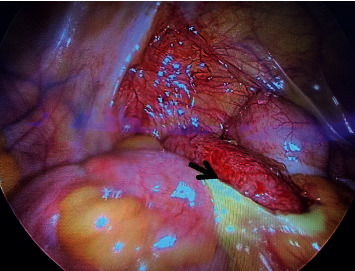
Intraoperative findings showing acutely inflamed appendix with fibrinous exudate.

The patient was conscious and had normal vital signs. Generally, was looking well, abdominal examination showed a right lower abdominal bruise, tenderness, rebound tenderness, and involuntary guarding in the right iliac fossa. Head to toe examination showed no other signs of trauma. Laboratory tests showed high inflammatory markers (white blood cell count (WBC) 15.5 K/*μ*L, hemoglobin 15.3 g/dL, platelets 207 K/*μ*L, CRP: 90.5, and bilirubin: 29.1). CT abdomen with IV and oral contrast was done and showed a dilated appendix in the right iliac fossa (16 mm in diameter), with wall enhancement and periappendiceal fat stranding (Figure 1). The patient was diagnosed with acute appendicitis, and an emergency laparoscopic appendectomy was performed. Intraoperative findings showed grossly inflamed appendix with fibrinous exudate with no collection or perforation (Figure 2), and the inspected other intra-abdominal solid and hollow organs were normal. Postoperatively, the patient recovered well and was discharged one day after surgery. On follow-up 2 weeks in the clinic, he was completely healthy, wounds were healed, and histopathology confirmed the diagnosis of acute appendicitis.

## 3. Discussion

The most commonly identified cause of acute appendicitis is the luminal obstruction leading to inflammation and complications of the appendix [2]. One of the earliest well-documented reports that linked blunt abdominal trauma (BAT) with traumatic appendicitis (TA) was the Hungarian stunt performer, Harry Houdini, who used to voluntarily hit his abdomen as a show of strength, subsequently developed peritonitis due to perforated appendix and died [3]. Despite the reports on the possible relationship between BAT and appendicitis are limited (Table 1), however, many theories support this relationship [4]. Some speculated that BAT might cause inflammation by the direct impact and appendiceal injury, and others attributed it to the indirect effect, leading to increased intraluminal pressure followed by burst or intraluminal pressure induced mucosal injury resulting in hematoma/edema that will cause luminal narrowing followed by obstruction and inflammation [4].

Looking at the demographic characteristics of patients who develop TA, most of the reported cases (including ours) showed male predominance, similar to nontraumatic appendicitis; however, in former, more male predominance is expected as blunt trauma is more frequent among males [5], mostly seen in pediatric age group in contrast to our case who was an adult (Table 1). A possible explanation underlying pediatric patients' predominance is the smaller abdominal cavity, softer, and less muscular abdominal wall as compared with adults, where the transmission of energy following trauma is more significant leading to greater increase in intra-abdominal pressure, causing increased appendicular luminal pressure and thus appendicitis. Older children may represent the most sensitive age group due to the fact that they are more independent to participate in risky outdoor activities than their younger counterparts [1].

Patients usually present as the classical picture of acute appendicitis with a difference of preceding trauma, developing abdominal pain within 6-48 hours following the severe blunt abdominal injury. This can be associated with other typical symptoms of acute appendicitis including nausea, vomiting, and anorexia (Table 1). Our patient had abdominal pain that started few hours after the BAT, in agreement with most of other reported cases in literature.

As for investigations, similar to that of nontraumatic appendicitis, blood tests usually show raised inflammatory markers and peculiar clinical signs and imaging, specifically CT scan of the abdomen, if required will confirm the diagnosis (Table 1), as our patient showed leukocytosis and had features of acute appendicitis on abdominal CT scan.

Diagnostic criteria for TA were postulated by Shutkin and Wetzler as follows:

1. Absolute freedom from abdominal symptoms, including pain, nausea, vomiting, and tenderness, before the trauma
2. Direct trauma must be severe and forcible, involving the abdominal wall and specially in the right half
3. Indirect trauma must be violent, acute, and unexpected
4. Symptoms must appear immediately after the trauma
5. Symptoms must be persistent and progressive, assuming the symptoms and signs of acute appendicitis
6. The pathologic findings must indicate a suppurative, destructive, or necrotic process [3]

Our patient fulfilled all the mentioned criteria, so we regarded it as TA.

As for the management, TA does not differ than nontraumatic appendicitis, with mainstay of treatment being surgical appendectomy (Table 1).

In terms of postoperative recovery, we noticed that patients treated for TA have relatively longer hospital stay than those with nontraumatic cause (Table 1). This might be because of the accompanied injuries; in fact, some of these patients presented with polytrauma, and TA was just a part of their multisystem involvement.

## 4. Conclusions

Blunt abdominal trauma caused by a camel kick to the abdomen requires a close observation of the patients. A camel kick may increase intra-abdominal pressure and cause indirect injury to internal organs including the appendix. Therefore, abdominal pain in these patients should not be regarded as being caused solely by abdominal wall contusion, and acute appendicitis should be considered in the differential diagnosis in any patient with abdominal pain following blunt abdominal trauma.

## Figures and Tables

**Table 1:** Summary of the case reports with publication year showing the cases of acute appendicitis secondary to traumatic abdominal injuries.

Study^∗^	Sex	Age	PH	MOI	Presentation	D	Examination	Labs	XR	US	CT	Surgery	Intraop	Histo	HS
Current study Qatar	M	35 y	UR	Camel kick	RLQ pain few hours after the kick, anorexia, V	2 d	Vit: Nr, RLQ bruise, tenderness	WBC: 15.5, CRP: 90.5, bilirubin: 29.1	NR	NR	Ap 16 mm, wall enhancement, periappendiceal fat stranding	L Appy	AA	AA	12 h
Zvizdic 2019 Bosnia & Herzegovina Cc [2]	M	7 y	UR	Horse kick	Sudden, progressive pain in RLQ, V	10 h	BP: 95/55, P: 110, T: 38.3, RR: 18. Abrasions, swelling tenderness and guarding RLQ	WBC: 11.5, Hb: 13.2, Plt: 280	NR	Pericecal free fluid, extending to pouch of Douglas	Small focus of free peritoneal air, free pelvic fluid	Lap, Appy	Perf AA with localized peritonitis	Perf AA, full thickness inflammation of Ap wall	6 d postop
Cobb 2017 U.S [6]	M	17 y	AD	MVC	Diffuse progressive Abd pain 24 h after MVC, V 10 times	NR	Vit: Nr, tender in both LQ's (Lt>Rt) and Lt upper quadrant	WBC: 10.8, Hb: 15.8, Plt: 243	Abd XR: abnormal bowel loop RLQ	NR	Dilated Ap 1.3 cm surrounding fluid in RLQ and pelvis	L Ex, Lap, Appy	Inflamed Ap, dark fluid in RLQ concerning for viscus injury	AA	10 d postop
Ahmed 2014 India [4]	M	12 y	UR	Hit disk's corner	Periumbilical Abd pain 1 d after trauma, fever	1 d	P: 114, BP: 90/56, minimal Abd movement with resp, bruise RLQ, rigid diffusely tender abdomen	WBC: 17, Hb: 10.5	Abd XR: air under diaphragm	Pelvic free fluid	NR	Ex Lap, Appy	Pus in pelvis, Perf AA at tip	AA	>4 d
Paschos 2012 Greece [7]	F	17 y	UR	Bicycle accident	Abd pain 12 h after trauma, discharged and came back with pain, anorexia, N&V	1 d	Vit: Nr, ecchymosis and tenderness RLQ	WBC: 9.1, on readmission: 12.7, Hb: 1.95 mmol/L	NR	Abd free fluid	NR	Lap, Appy	Free fluid, AA, contusion cecal base	AA	2 d
Torres-Grau 2012 UK [3]	M	15	UR	Fall from bicycle	Abd pain 30 m after fall	6 h	BP: 105/47, P: 57, T: 36.7, RLQ tenderness	WBC: 16.2, Neu: 13.6, Hb: 14.4, CRP: 1 Amy	NR	Free fluid in the peritoneum	NR	L Ex, Appy	Necrotic, non-Perf Ap	AA	NR
Atalla 2010 Australia [8]	M	53 y	UR	Fall on edge of car door	Abd pain 7 h after fall	7 h	P: ↑, BP: ↓, RLQ tenderness and guarding	WBC: ↑	NR	UR	Thickened Ap (10 mm) with fat stranding	L Ex, Appy	AA	AA	1 d postop
Toumi 2010 UK [1]	M	11 y	UR	Injury by elbow to RLQ	Abd pain after trauma, N&V, anorexia, fever	3 d	T: ↑, P: ↑, RLQ tenderness	UA: trace blood; Inf m: ↑	NR	NR	AA with adjacent collection	Appy	AA	AA serositis	4 d postop
Etensel 2005 Turkey [9]	M	9 y	NR	Fall	Polytrauma	1 h	BP: 80/50, P: 86, T: 36.7, confused, resp dist, head and Lt chest abrasions, ↓ breath sounds Lt chest	Hb: 11.2, WBC: 17.2	Chest and Abd XR: Lt lung contusion, free peritoneal air	Free air	Head CT: brain edema, Lt parietal bone fx; Abd CT: free air	Lap, Appy	AA, no bowel Perf	AA	10 d
Houry 2001 Colarado [10].	M	5 y	UR	Fall	Abd pain	1 h	BP: 114/75, P: 149, RR:32, T: 37.7, Abd tenderness and guarding more in RLQ	N	N	Free fluid in pouch of Douglas	Pelvic free fluid, AA	Ex Lap, Appy	AA, Perf at the base	AA	NR
Serour 1996 Israel [11]	M	11 y	UR	Punch	RLQ pain, N&V	18 h	BP: 115/60, P: 100, T: 37, looked ill, RLQ tenderness	Hb: 13.7, WBC: 4.5	NR	NR	Calcified appendicolith, prerectal fluid	Lap. Appy	Gangrenous Ap	Gangrenous appendicitis with periappendicitis	NR
Serour 1996 Israel [11]	M	8 y	UR	Fall	Abd pain, N and fever	3 h	BP: 95/55, P: 96, T: 38.2, ecchymosis over right side of the face, RLQ tenderness, guarding, and rebound	Hb: 12.5, WBC: 20.1	NR	NR	NR	Appy	AA	Phlegmonous appendicitis with periappendicitis	NR
Serour 1996 Israel [11]	M	7 y	UR	Fight	Abd pain, fever, V	Few d	T: 40, acute Abd	NR	NR	NR	Abscess in RLQ	Appy, drainage of abscess	Gangrenous Perf Ap	Gangrenous Perf appendicitis	NR
Ciftci 1996 Turkey [12]	M	8	NR	MVC	Abd pain	2 h	NR	NR	NR	NR	NR	Appy	Perf Ap	AA	Average hospital stay 6.4 d
Ciftci 1996 Turkey [12]	F	5	NR	Fall	Abd pain	6 h	NR	NR	NR	NR	NR	Appy	AA	AA	
Ciftci 1996 Turkey [12]	F	13	NR	Ball	N	12 h	NR	NR	NR	NR	NR	Appy	AA	AA	
Ciftci 1996 Turkey [12]	M	14	NR	MVC	Abd pain	4 h	NR	NR	NR	NR	Dilated bowel loops, free fluid	Appy	Perf Ap	AA	
Ciftci 1996 Turkey [12]	M	7	NR	Assault	Abd pain	12 h	NR	NR	NR	NR	Dilated bowel loops, free fluid	Appy	AA	AA	

^∗^For space considerations, only the first author is cited; AA: acute appendicitis; Abd: abdomen/al; AD: atopic dermatitis; Ap: appendix/appendiceal; Appy: appendectomy; BP: blood pressure in mmHg; CRP: C-reactive protein; D: duration of symptoms; d: days; diaph: diaphragm/atic; Ex: exploratory/ion; fx: fracture; h: hour/s; Hb: hemoglobin g/dL; Hem: hematoma; Histo: histology; HS: hospital stay postoperatively; Intraop: intraoperative findings; Inf m: inflammatory markers; I.O: intestinal obstruction; L: laparoscopic; Lap: laparotomy; LQ: lower quadrant; Lt: left; M: male; m: month/s; min: minute/s; McB: McBurney's point; MOI: mechanism of injury; MVC: motor vehicle collision; N: nausea; Neu: neutrophils; NR: not reported; Nr: normal; P: pulse in beats per minute; Perf: perforation; PH: past history; pneumomed: pneumomediastinum; Plt: platelets K/*μ*L; postop: postoperative; resp: respiration; Retro: retroperitoneal; resp dist: respiratory distress; RLQ: right lower quadrant; RR: respiratory rate; Rt: right; T: temperature °C; UA: urine analysis; UR: unremarkable; V: vomiting; Vit: vital signs; WBC: white blood cell count K/*μ*L; y: year/s; ↑: high; ↓: low.

## Data Availability

The data used to support the findings of this study are included in the article.
